# STING-induced noncanonical autophagy regulates endolysosomal homeostasis

**DOI:** 10.1073/pnas.2415422122

**Published:** 2025-02-21

**Authors:** Tuozhi Huang, Chenglong Sun, Fenghe Du, Zhijian J. Chen

**Affiliations:** ^a^Department of Molecular Biology, University of Texas, Southwestern Medical Center, Dallas, TX 75390-9148; ^b^Center for Inflammation Research, University of Texas, Southwestern Medical Center, Dallas, TX 75390-9148; ^c^HHMI, University of Texas, Southwestern Medical Center, Dallas, TX 75390-9148

**Keywords:** cGAS, STING, autophagy, TFEB, ESCRT

## Abstract

The cGAS–STING pathway mediates innate immune responses to cytosolic DNA. In addition to its well-established role in inducing inflammatory cytokines, activation of the cGAS–STING pathway also induces noncanonical autophagy, a process involving the conjugation of the ATG8 family of ubiquitin-like proteins to membranes of the endolysosomal system. The mechanisms and functions of STING-induced autophagy remain poorly understood. In this study, we demonstrated that STING activation induced formation of pH-elevated Golgi-derived vesicles that led to ATG16L1 and V-ATPase-dependent noncanonical autophagy. We showed that STING-induced noncanonical autophagy resulted in activation of the MiT/TFE family of transcription factors (TFEB, TFE3, and MITF), which regulate lysosome biogenesis. We found that lipidation of the ATG8 proteins, particularly GABARAPs, inhibited phosphorylation of MiT/TFE transcription factors by mTORC1. The lipidated GABARAPs bound to the Folliculin-interacting proteins (FNIPs), thereby sequestering the FNIP-folliculin protein complexes from activating mTORC1, resulting in dephosphorylation and nuclear translocation of MiT/TFE transcription factors. Furthermore, we found that STING-induced autophagy activated Leucine-rich repeat kinase 2 (LRRK2), a protein implicated in Parkinson’s disease, through GABARAPs lipidation. We further showed that STING-induced autophagy induced ALIX-mediated ESCRT machinery recruitment to mitigate endolysosomal perturbation. These results reveal the multifaceted functions of STING-induced noncanonical autophagy in regulating endolysosomal homeostasis.

## Significance

The cGAS–STING pathway plays a key role in innate immunity by producing proinflammatory cytokines in response to cytosolic DNA. Moreover, activation of the pathway also leads to noncanonical autophagy, an evolutionarily conserved process with poorly understood mechanism and function. Here, we showed that STING trafficking to Golgi-derived vesicles elevated the pH in these vesicles, resulting in the lipidation of these vesicles by the ATG8 family of ubiquitin-like proteins. STING-induced autophagy promotes endolysosomal biogenesis through activation of the MiT/TFE transcription factors and the kinase LRRK2, which has been linked to Parkinson’s disease. We further showed that STING-induced autophagy recruits the ESCRT machinery to facilitate membrane damage repair. These results uncover a role of the cGAS–STING pathway in regulating lysosome homeostasis.

The cyclic GMP-AMP synthase (cGAS)–Stimulator of interferon genes (STING) pathway plays a pivotal role in innate immunity by detecting cytosolic DNA and initiating proinflammatory cascades (1–5). Upon binding to double-stranded DNA, cGAS catalyzes the synthesis of 2′3′-Cyclic-GMP-AMP (cGAMP) which serves as a second messenger to activate STING (4, 5). Once activated, STING, an endoplasmic reticulum (ER)-localized protein, translocates from the ER through the Golgi to lysosomes. During its trafficking, STING recruits and activates TANK-binding kinase 1 (TBK1) and IκB kinase (IKK) via its C-terminal tail, leading to activation of the transcription factor IRF3 and NF-κB, which subsequently turn on the production of type I interferons and inflammatory cytokines (6–8).

cGAMP-induced trafficking of STING also induces robust conjugation of lipid membranes by the ATG8 family of ubiquitin-like proteins, including microtubule-associated protein light chain 3 proteins (LC3A, LC3B, and LC3C) and the gamma-aminobutyric acid type A receptor-associated proteins (GABARAP, GABARAPL1, and GARBARAPL2) (9–11). Unlike canonical autophagy, which employs the ULK1/ULK2 protein kinases and VPS34 lipid kinase complexes to initiate LC3 lipidation, STING-induced LC3 lipidation does not require ULK1 or VPS34 but requires the lysosomal V-ATPase (9, 11). The ability to induce this noncanonical autophagy is a primordial function of STING that is conserved in metazoan animals (9), but the physiological function of STING-induced noncanonical autophagy remains poorly understood.

In this study, we found that an important function of STING-induced noncanonical autophagy is to activate the microphthalmia/transcription factor E (MiT/TFE) family of transcription factors, which regulate lysosome biogenesis. The MiT/TFE family consists of four members: microphthalmia-associated transcription factor (MITF), transcription factor EB (TFEB), transcription factor binding to IGHM enhancer 3 (TFE3), and transcription factor EC (TFEC) (12). MiT/TFE transcription factors serve as master regulators of autophagy and endolysosomal biogenesis through binding to coordinated lysosomal expression and regulation (CLEAR) motif in the promoters of many lysosome- and autophagy-related genes (13–15). These transcription factors are predominantly regulated by phosphorylation. Phosphorylated TFEB, TFE3, and MITF are sequestered in the cytosol by the chaperone protein 14-3-3 (15–19). Upon endolysosomal damage, TFEB, TFE3, and MITF undergo dephosphorylation which enables their translocation into the nucleus to initiate endolysosomal biogenesis programs (18–22).

Leucine-rich repeat kinase 2 (LRRK2) is a Rab-GTPase kinase implicated in maintaining cellular homeostasis in response to disruption of the Golgi network and the endolysosomes (23–25). Upon activation, LRRK2 phosphorylates a subgroup of RAB GTPases, such as Rab8/10/12, to promote lysosomal cargo secretion and suppress lysosomal enlargement (23, 25). Mutations of LRRK2 have been linked to Parkinson’s disease and Crohn’s disease. In this study, we found that STING trafficking activates LRRK2 through GABARAPs lipidation.

In addition, we found that STING-induced noncanonical autophagy led to the recruitment of ALG-2-interacting protein X (ALIX) to the STING vesicles. ALIX plays an important role in recruiting endosomal sorting complexes required for transport (ESCRT) machinery to maintain endolysosomal homeostasis (26–28). In response to endolysosomal membrane perturbation, ALIX facilitates ESCRT machinery assembly through recruiting ESCRT-III proteins onto the damaged sites to promote membrane invagination, which subsequently leads to shedding of damaged membrane into the lumen of endolysosomes, resulting in membrane repair (26–29). Altogether, our findings unveil key functions of STING-induced noncanonical autophagy in regulating endolysosomal homeostasis.

## Results

### STING Activation Elevates pH in Golgi-Derived Vesicles, Leading to LC3 Lipidation Through V-ATPase and ATG16L1.

**Fig. 1.**
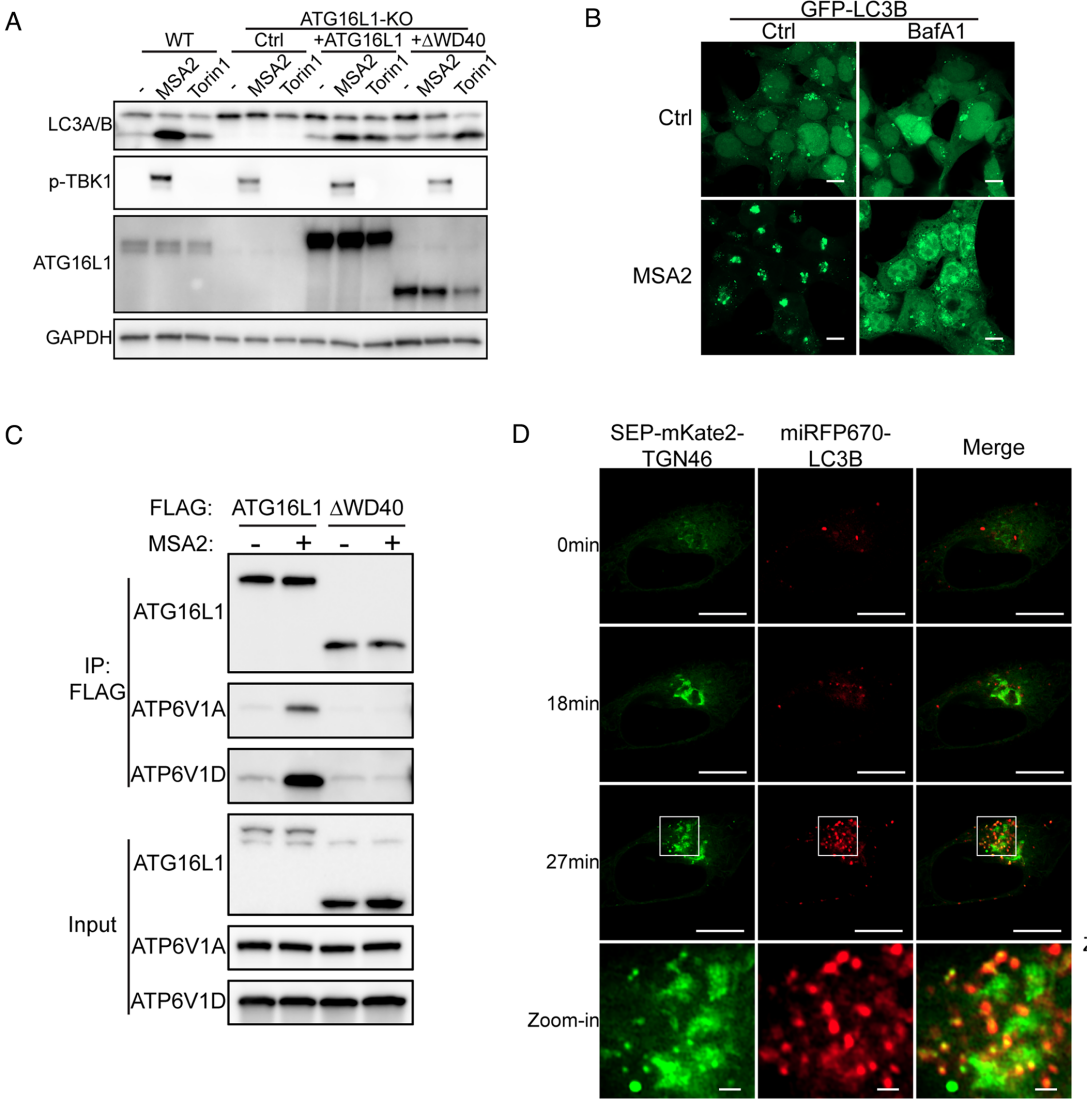
STING activation elevates pH in Golgi-derived vesicles, leading to LC3 lipidation through V-ATPase and ATG16L1. (*A*) HeLa-STING(WT), HeLa-STING-ATG16L1KO, HeLa-STING-ATG16L1, and HeLa-STING-ATG16ΔWD40 cells were stimulated with 30 µM MSA2 for 2 h, or 100 nM Torin1 for 5 h, or untreated. The cell lysates were analyzed by immunoblotting as indicated. (*B*) HEK293T FIP200-KO cells stably expressing STING and GFP-LC3B were stimulated with indicated compounds (30 µM MSA2, 100 nM bafilomycin A1) for 2 h, or untreated. Cells were analyzed by confocal microscopy. (Scale bar, 10 µm.) (*C*) HeLa-STING-ATG16L1 and HeLa-STING-ATG16ΔWD40 cells were stimulated with 30 µM MSA2 for 1.5 h or untreated. Protein complexes were immunoprecipitated with a Flag antibody and analyzed by immunoblotting. (*D*) HeLa cells stably expressing STING, miRFP670-LC3B, and Super Ecliptic pHluorin (SEP)-mKate2-TGN46 were treated with 1 µM diABZI, and the cells were analyzed by live cell microscopy. Representative images taken at 0 min, 18 min, and 27 min are shown. The *Left* panels show the SEP signal of SEP-mKate2-TGN46 fusion protein. (Scale bar, 10 µm) for images on the *Upper* panels and 1 µm for Zoom-in images on the *Bottom* panels.

Recent studies have shown that V-ATPase plays an important role in noncanonical autophagy including LC3-associated phagocytosis (LAP) and Xenophagy (30–34). Consistent with a previous report (11), we found that stimulation of HeLa cells stably expressing STING with the STING agonist MSA2 (35) led to robust lipidation of LC3 in a manner that depended on the WD40 domain of ATG16L1 (Fig. 1*A*). In contrast, the WD40 domain of ATG16L1 was dispensable for LC3 lipidation induced by Torin1, a compound that induces canonical autophagy through inhibition of mTORC1 (Fig. 1*A*).

To test whether V-ATPase is required for STING-induced noncanonical autophagy, we generated a 293T cell line stably expressing STING and GFP-LC3B. In this cell line, we further employed CRISPR to delete the gene encoding FIP200, an essential gene for macroautophagy (36), to reduce basal autophagy flux. Upon MSA2 treatment, GFP-LC3B puncta formation was observed, indicating LC3B lipidation (Fig. 1*B*). V-ATPase inhibitor bafilomycin A1 (BafA1) treatment abolished the GFP-LC3B puncta formation, suggesting a critical role of V-ATPase in STING-induced noncanonical autophagy (Fig. 1*B*).

To determine whether STING activation induces the interaction between V-ATPase and ATG16L1, we performed immunoprecipitation in HeLa-STING-ATG16L1 cells and HeLa-STING-ATG16ΔWD40. Endogenous V-ATPase subunit V1A and V1D were coimmunoprecipitated with full-length ATG16L1 in the cells stimulated with MSA2, whereas such interaction was lost in ATG16ΔWD40 cells (Fig. 1*C*), suggesting that STING activation induces the interaction between ATG16L1 and V-ATPase in a manner that depends on the WD40 domain of ATG16L1.

V-ATPase pumps proton into intracellular membrane vesicles, including the Golgi, endosome, and lysosome, to maintain a low pH in the lumen of these vesicles. It was recently reported that STING functions as a proton channel to raise the pH of the Golgi or post-Golgi vesicles (37, 38). The elevated pH within these vesicles may serve as a signal to activate the V-ATPase, which then recruits ATG16L1 to initiate noncanonical autophagy. To test this model, we generated a series of pH sensors in the secretory pathway. Briefly, superecliptic pHluorin [SEP, a GFP variant that can be quenched at low pH (39, 40)] and mKate2(a far-red fluorescence protein insensitive to pH change) were fused to trans-Golgi localized proteins TGN46 and B4GALT1 (SEP-mKate2-TGN46 and B4GALT1-SEP-mKate2), and transferrin receptor (TfR- SEP-mKate2), which distributes across the entire secretory pathway. Higher pH will lead to an increase of SEP signal intensity while mKate2 serves as an internal control. Consistent with previous reports (37, 38), we found that stimulation of HeLa cells stably expressing STING with the STING agonist diABZI (41) led to pH increase in the Golgi compartments (Fig. 1*D* and SI Appendix, Fig. S1 A–C). To delineate the STING vesicles modified by LC3, we generated a HeLa cell line stably expressing STING, miRFP670-LC3B, and SEP-mKate2-TGN46. We found that diABZI treatment induced lipidation of miRFP670-LC3B shortly after the pH increase in the Golgi (Fig. 1*D* and Movie S1). Upon closer examination, we found that STING activation induced formation of pH-elevated Golgi-derived vesicles. miRFP670-LC3B exclusively colocalized with these Golgi-derived vesicles, but not with the main body of the Golgi (Fig. 1*D* and Movie S1). Taken together, these data suggest that STING activation induces LC3 lipidation on pH-elevated Golgi-derived vesicles through the V-ATPase–ATG16L1 pathway (SI Appendix, Fig. S1D).

### Noncanonical Autophagy Is Required for STING-Induced Activation of MiT/TFE Transcription Factors to Regulate Endolysosomal Biogenesis.

**Fig. 2.**
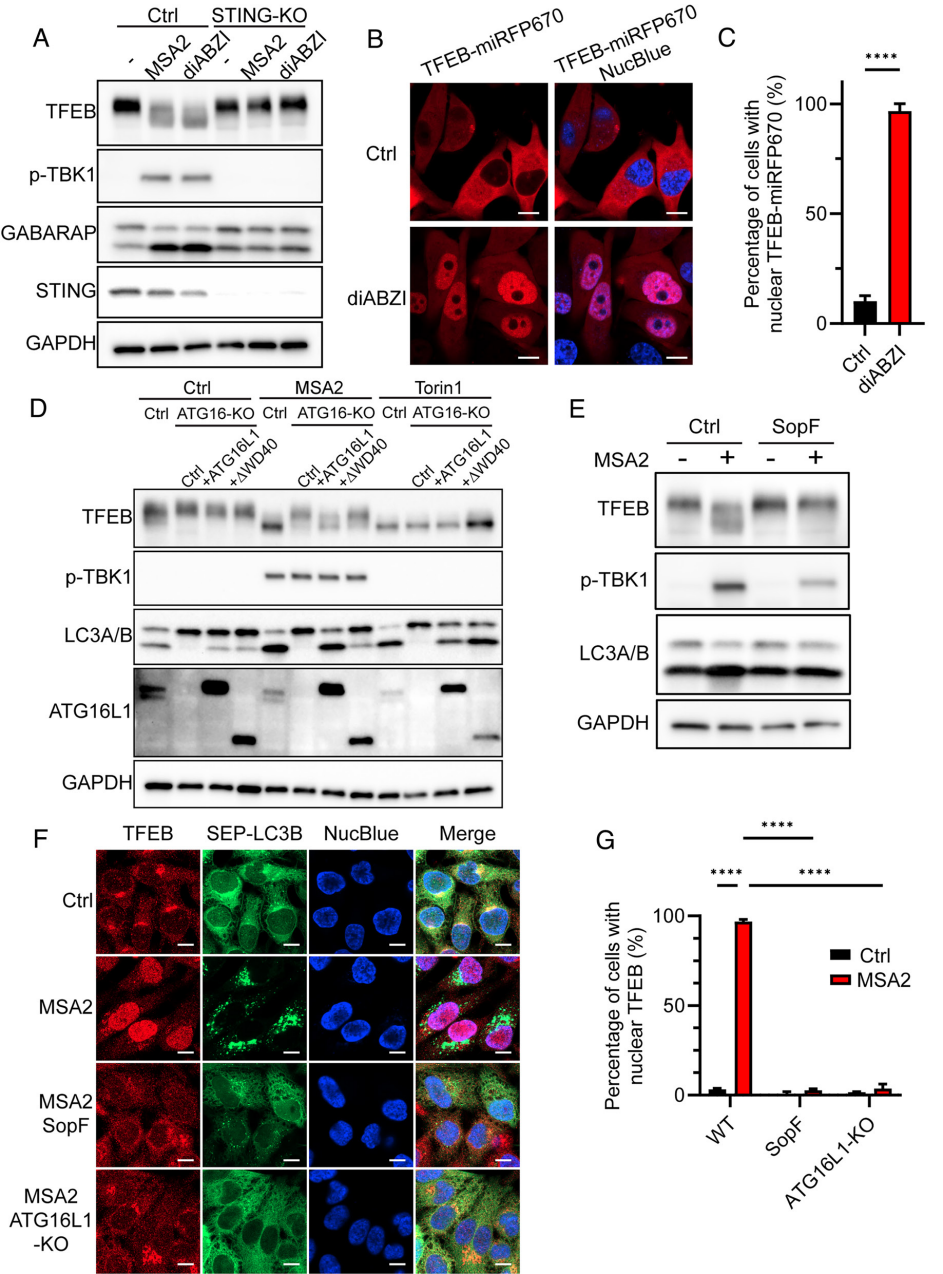
Noncanonical autophagy is required for STING-induced activation of MiT/TFE transcription factors. (*A*) HeLa-STING (ctrl) and HeLa-STING-KO cells were treated with 30 µM MSA2 or 50 nM diABZI for 2 h or untreated, followed by analyses of cell lysates by immunoblotting with the indicated antibodies. (*B*) HeLa-STING cells stably expressing TFEB-miRFP670 were treated with 50 nM diABZI for 2 h or untreated, and live cells were imaged by microscopy. (Scale bar, 10 µm.) (*C*) Quantification of 77 and 80 cells per group as represented by *B*. Error bars represent SD. Tested by unpaired Student’s *t* test. ****P < 0.0001. (*D*) HeLa-STING, HeLa-STING-ATG16L1KO, HeLa-STING-ATG16L1, and HeLa-STING-ATG16ΔWD40 cells were stimulated with 30 µM MSA2 for 2 h or 100 nM Torin1 for 5 h or untreated. The cell lysates were analyzed by immunoblotting as indicated. (*E*) HeLa cells stably expressing STING (ctrl) or both STING and SopF (SopF) were treated with 30 µM MSA2 for 2 h or untreated. The cell lysates were analyzed by immunoblotting. (*F*) HeLa-STING, HeLa-STING-SopF, and HeLa-STING-ATG16L1KO cells stably expressing SEP-LC3B were treated with 30 µM MSA2 for 2 h or untreated. The cells were stained with an antibody against endogenous TFEB followed by confocal microscopy analyses. (Scale bar, 10 µm.) (*G*) Quantification of 88 to 228 cells per group as represented by *F*. Error bars represent SD. Tested by Tukey’s multiple comparisons test. ****P < 0.0001.

Our finding that STING activation and trafficking lead to LC3 lipidation on post-Golgi endosomes suggests that the LC3 lipidation may regulate endolysosome homeostasis. A master regulator of lysosome biogenesis is the MiT/TFE family of transcription factors (42). Indeed, we observed that TFEB was dephosphorylated upon stimulation by MSA2 or diABZI in HeLa cells stably expressing STING (HeLa-STING) (Fig. 2*A*). Both endogenous TFEB and TFEB-miRFP670 fusion protein were transported into the nucleus as a result of their dephosphorylation in the cells stimulated with MSA2 or diABZI (Fig. 2 *B*, *C*, *F* and *G*). Two other members of the MiT/TFE family, TFE3 and MITF, also translocated into the nucleus upon stimulation, whereas TFEC showed constitutive nuclear localization (SI Appendix, Fig. S2). Together, these data demonstrated that STING activation leads to the dephosphorylation and nuclear translocation of MiT/TFE transcription factors.

To test whether STING-induced autophagy plays a role in activating the MiT/TFE pathway, we treated HeLa-STING cells, HeLa-STING-ATG16L1 knockout (KO) cells, HeLa-STING-ATG16L1 cells, and HeLa-STING-ATG16ΔWD40 cells with MSA2 or Torin1. Remarkably, ATG16L1 KO abolished STING-induced TFEB dephosphorylation in HeLa-STING cells, which could be rescued by full-length ATG16L1 but not ATG16ΔWD40 (Fig. 2*D*). In contrast, Torin1 treatment led to TFEB dephosphorylation in all cells (Fig. 2*D*).

SopF is a bacterial effector protein that suppresses noncanonical autophagy by disrupting the interaction between V-ATPase and ATG16L1 (33, 43). Stable expression of SopF inhibited TFEB dephosphorylation after MSA2 treatment in HeLa-STING cells (Fig. 2*E*). In line with this, both SopF ectopic expression and deletion of ATG16L1 were sufficient to inhibit the nuclear translocation of TFEB (Fig. 2 *F* and *G*), TFE3, and MITF (SI Appendix, Fig. S2).

**Fig. 3.**
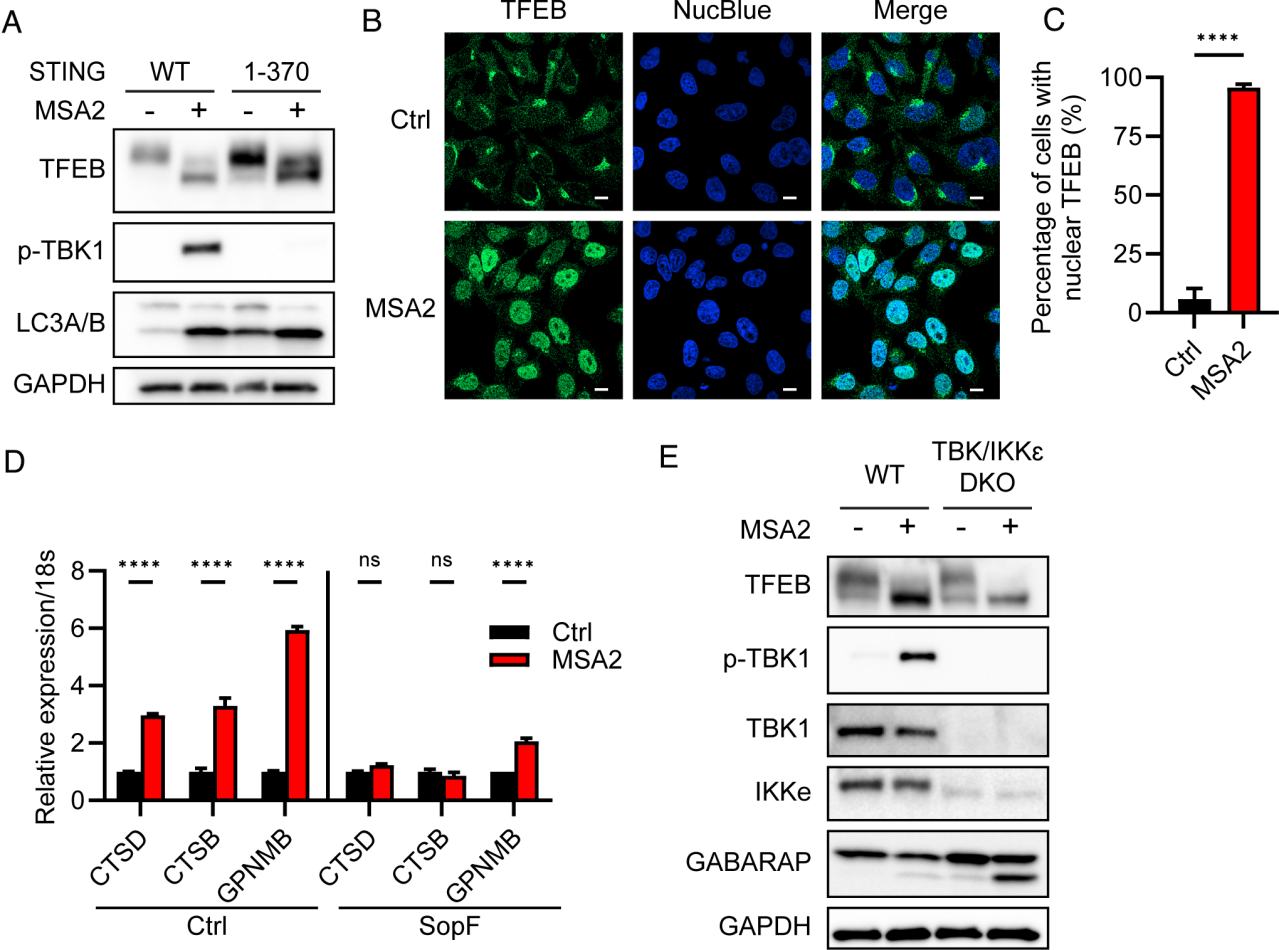
TFEB activation by STING does not require TBK1. (*A*) HeLa-STING and HeLa-STING (1 to 370) cells were treated with 30 µM MSA2 for 2 h or untreated. Cell lysates were analyzed by immunoblotting. (*B*) HeLa-STING (1 to 370) cells were stimulated with 30 µM MSA2 for 2 h or untreated; then, cells were imaged by confocal microscopy using an antibody against TFEB. NucBlue stains nuclear DNA. (Scale bar, 10 µm.) (*C*) Quantification of 67 and 77 cells per group as represented by *B*. Error bars represent SD. Tested by unpaired Student’s *t* test. ****P < 0.0001. (*D*) HeLa-STING (1 to 370), and HeLa-STING (1 to 370)-SopF cells were treated with 10 µM MSA2 for 24 h or untreated. RNA was isolated from these cells for analysis by quantitative RT-PCR to measure the expression of indicated TFEB target genes. CTSD: cathepsin D; CTSB: cathepsin B; GPNMB: Glycoprotein Nonmetastatic Melanoma Protein B. The mRNA expression levels of the MSA-treated cells were normalized to untreated cells (ctrl). Error bars represent SD. The data are representative of 2 independent experiments. Tested by Tukey’s multiple comparisons test. ****P < 0.0001. (*E*) wild-type (WT) and TBK1/IKKε double knockout (DKO) THP1 cells were treated with 30 µM MSA2 for 2 h or untreated. Then cell lysates were analyzed by immunoblotting.

We have previously shown that STING-induced noncanonical autophagy can be uncoupled from STING-induced TBK1 activation because deletion of the STING C-terminal tail abolished TBK1 activation but not LC3 lipidation (9). Consistent with this finding, a STING C-terminal-truncated mutant [STING (1-370)] still induced TFEB dephosphorylation and nuclear translocation after MSA2 stimulation despite its inability to stimulate TBK1 phosphorylation (Fig. 3 *A*–*C*). Stimulation of this STING mutant cell line increased the expression of MiT/TFE target genes, which could be suppressed by SopF (Fig. 3*D*). Furthermore, TFEB still underwent dephosphorylation upon MSA2 treatment in THP1 cells lacking both TBK1 and IKKε, which are required for IRF3 and NF-κB activation (Fig. 3*E*).

**Fig. 4.**
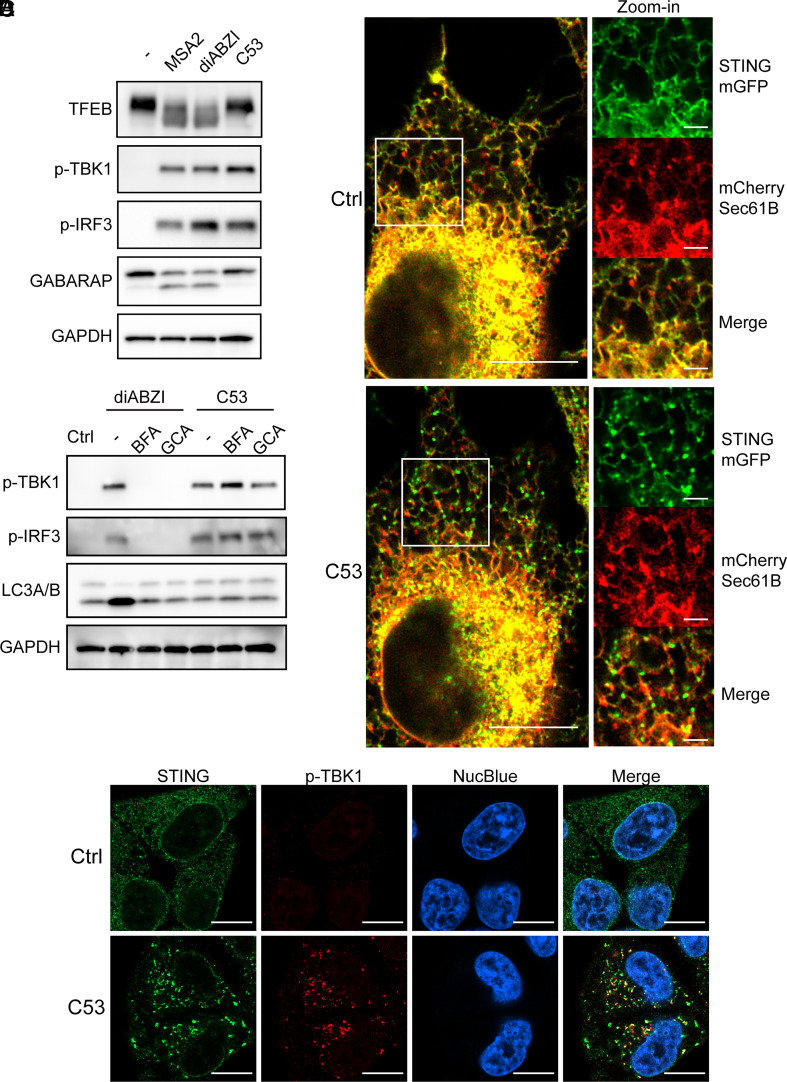
STING agonist C53 activates TBK1 and IRF3, but not TFEB, through a mechanism independent of membrane trafficking. (*A*) HeLa-STING cells were treated with 30 µM MSA2, 50 nM diABZI, or 10 µM C53 for 2 h or untreated, then cell lysates were analyzed by immunoblotting. (*B*) HeLa STING-KO cells stably expressing STING-mGFP and mCherry-Sec61B were treated with 1.25 µM C53, and the cells were analyzed by live cell microscopy. Representative images taken at 0 min (Ctrl) and 10 min (C53) are shown. (Scale bar, 10 µm) for images on the *Left* panels and 2 µm for Zoom-in images on the *Right* panels. (*C*) HeLa-STING cells were treated with 1 mM BFA or 10 mM GCA for 20 min before stimulation with 50 nM diABZI or 10 µM C53 for 2 h. Cell lysates were analyzed by immunoblotting with indicated antibodies. (*D*) HeLa-STING cells were stimulated with 1.25 µM C53 for 20 min or untreated, then cells were imaged by confocal microscopy using antibodies against STING and p-TBK1. NucBlue stains nuclear DNA. (Scale bar, 10 µm.)

We also treated the cells with C53, a recently discovered STING agonist that activates proinflammatory cascades without triggering noncanonical autophagy (37, 38, 44). C53 treatment in HeLa-STING cells failed to induce TFEB activation despite strong phosphorylation of TBK1 and IRF3 (Fig. 4*A*). Interestingly, C53 treatment led to rapid formation of STING puncta that colocalized with the ER membrane marker Sec61B (Fig. 4*B* and Movie S2) and phospho-TBK1 (Fig. 4*D*). Treatment of cells with brefeldin A (BFA) or golgicide A (GCA), both of which inhibit membrane trafficking from the ER to Golgi, inhibited phosphorylation of TBK1 and IRF3 in response to diABZI but not C53, suggesting that C53 activates TBK1 and IRF3 by promoting aggregation of STING on the ER membrane without causing STING to traffic from the ER to Golgi (Fig. 4*C*). These results show that once STING is oligomerized, its trafficking from the ER to Golgi is no longer required for the activation of TBK1 or IRF3. In line with this, a recent study showed that forced oligomerization of the C-terminal tail of STING in the cytosol is sufficient to induce TBK1 activation (45). Taken together, these results indicate that TFEB activation requires STING trafficking-induced noncanonical autophagy but not activation of TBK1 or IRF3.

### FNIP1/FNIP2–GABARAPs Interactions Mediate the Activation of MiT/TFE by STING.

**Fig. 5.**
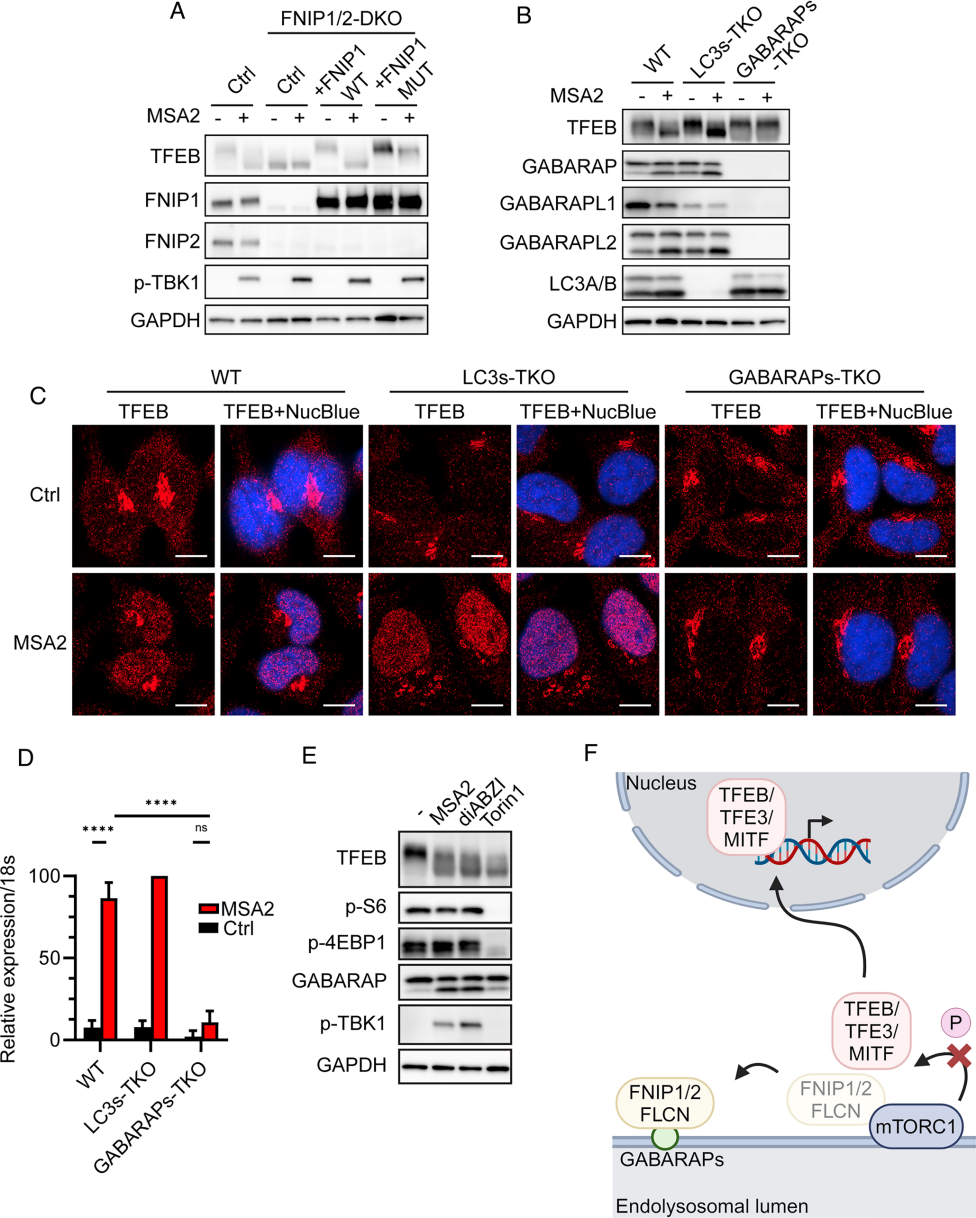
FNIP1/FNIP2–GABARAP interactions mediate the activation of TFEB by STING. (*A*) HeLa-STING (Ctrl), HeLa-STING-FNIP1/2DKO, HeLa-STING-FNIP1WT, and HeLa-STING-FNIP1MUT cells were treated with 30 µM MSA2 for 2 h or untreated. Cell lysates were analyzed by immunoblotting as indicated. (*B*) HeLa-STING (WT), HeLa-STING-LC3sTKO, and HeLa-STING-GABARAPsTKO cells were treated with 30 µM MSA2 for 2 h or untreated. Cell lysates were analyzed by immunoblotting. (*C*) Similar to (*B*) except that cells were analyzed by immunofluorescent microscopy using an antibody against TFEB. TFEB+NucBlue: merging of TFEB staining with nuclear DNA staining by NucBlue. (Scale bar, 10 µm.) (*D*) Quantification of 46 to 76 cells per group as represented by *C*. Error bars represent SD. Tested by Tukey’s multiple comparisons test. ****P < 0.0001. (*E*) HeLa-STING cells were treated with 30 µM MSA2 or 50 nM diABZI for 2 h, or 100 nM Torin1 for 5 h, or untreated. Cell lysates were analyzed by immunoblotting. (*F*) Diagram depicting the mechanism by which STING-induced noncanonical autophagy promotes endolysosomal biogenesis through inhibiting the phosphorylation of TFEB, TFE3, and MITF by mTORC1.

Recent studies have shown that a complex consisting of Folliculin (FLCN) and Folliculin-interacting protein 1 or 2 (FNIP1/FNIP2) is required for phosphorylation of MiT/TFE transcription factors by mTORC1 (46, 47). Consistent with this, deletion of FNIP1 and FNIP2 in HeLa-STING cells led to constitutive dephosphorylation of TFEB (Fig. 5*A*). Stable expression of wild-type FNIP1 in the FNIP1/2 DKO cells restored the phosphorylation of TFEB, which is dephosphorylated in response to MSA2 stimulation (Fig. 5*A*). These results suggest that STING activation leads to TFEB dephosphorylation in a manner that depends on FNIP1/2.

FNIP proteins have been found to interact with GABARAPs, and this interaction disrupts the interaction between FLCN/FNIPs and mTORC1, thereby inhibiting TFEB phosphorylation by mTORC1 (48). We found that a FNIP1 mutant previously found to be defective in binding GABARAPs failed to support MSA2-induced dephosphorylation of TFEB (Fig. 5*A*) (48), indicating that the interaction between GABARAPs and FNIP1 impaired the phosphorylation of TFEB. To test which members of the ATG8 family are important for STING-induced TFEB activation, we used CRISPR to knock out three genes encoding GABARAPs (GABARAP1, GABARAPL1, and GABARAPL2) or three genes encoding LC3 (LC3A, LC3B, and LC3C) in HeLa-STING cells. MSA2 stimulation led to dephosphorylation of TFEB in the cells lacking the LC3s but not GABARAPs, supporting a unique role of GABARAPs in regulating TFEB (Fig. 5 *B*–*D*). These results suggest that STING-induced lipidation of GABARAPs leads to the recruitment of FLCN/FNIPs away from mTORC1, leading to TFEB dephosphorylation (Fig. 5*F*). Interestingly, STING stimulation does not lead to general inhibition of mTORC1 kinase activity, because Torin1, but not MSA2, inhibited the phosphorylation of the ribosomal subunit S6 or the translational regulator 4EBP1 by mTORC1 (Fig. 5*E*). Collectively, these results support a model in which STING-induced lipidation of GABARAPs sequesters the FNIP/FLCN complex from mTORC1, thereby inhibiting the phosphorylation of MiT/TFE family of transcription factors, resulting in their nuclear translocation and subsequent regulation of lysosome biogenesis (Fig. 5*F*).

### STING-Induced Noncanonical Autophagy Activates LRRK2.

**Fig. 6.**
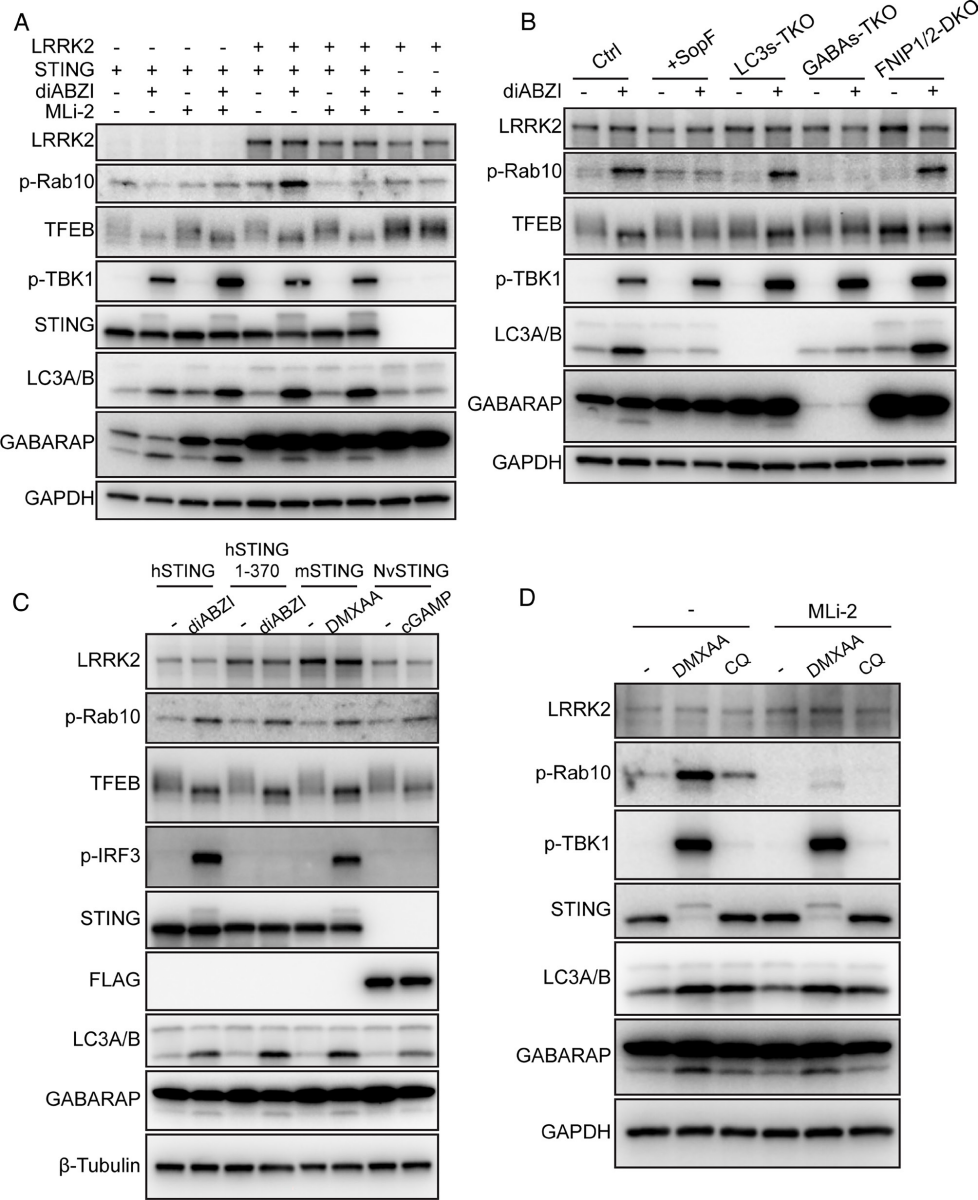
STING-induced noncanonical autophagy activates LRRK2. (*A*) HeLa cells stably expressing STING, LRRK2, or both were treated with indicated compounds (100 nM diABZI, 100 nM MLi-2) for 2 h. Cell lysates were analyzed by immunoblotting. (*B*) HeLa-STING (Ctrl), HeLa-STING-SopF (+SopF), HeLa-STING-LC3s TKO (LC3s-TKO), HeLa-STING-GABARAPs TKO (GABAs-TKO), and HeLa-STING-FNIP1/2 DKO (FNP1/2-DKO) cells were treated with 100 nM diABZI for 2 h or untreated. Cell lysates were analyzed by immunoblotting. (*C*) HeLa cells stably expressing human STING (*h*STING), C-terminal tail truncated human STING (*h*STING 1 to 370), murine STING (*m*STING), or FLAG tagged sea anemone STING (*Nv*STING) were treated with indicated STING agonists [100 nM diABZI, 20 ug/mL DMXAA, 10 μM cGAMP (following digitonin treatment)] for 2 h or untreated. Cell lysates were analyzed by immunoblotting. (*D*) BV-2 cells were treated with indicated compounds (20 µg/mL DMXAA, 100 µM Chloroquine (CQ, a known LRRK2 agonist), 100 nM MLi-2), or untreated for 2 h. Cell lysates were analyzed by immunoblotting.

Recent research showed that LRRK2 could be activated in a manner that depended on V-ATPase and ATG16L1 (49). We therefore tested whether STING-induced noncanonical autophagy could trigger LRRK2 activation. We found that LRRK2 expression was undetectable in Hela-STING cells, and treatment of these cells with the STING agonist diABZI was not able to induce phosphorylation of Rab10, a LRRK2 substrate (Fig. 6*A*). However, after stably expressing LRRK2 in Hela-STING cells, STING activation by diABZI led to significant phosphorylation of Rab10, which was blocked by the LRRK2 kinase inhibitor MLi-2 (Fig. 6*A*). LRRK2 activation was not detected in STING-deficient cells (Fig. 6*A*). Consistent with this, in BV-2 cells, a murine microglial cell line with relatively high LRRK2 expression, STING activation by DMXAA (a murine STING agonist) led to robust phosphorylation of Rab10, which was inhibited by MLi-2 (Fig. 6*D*).

To further investigate the mechanism of LRRK2 activation by STING, LRRK2 was introduced into various engineered HeLa cell lines (Fig. 6 *B* and *C*). diABZi induced Rab10 phosphorylation in the HeLa-STING cells lacking LC3s, but not GABARAPs (Fig. 6*B*). Overexpression of SopF inhibited Rab10 phosphorylation in response to diABZI (Fig. 6*B*). In HeLa-*h*STING (1 to 370) cells in which the C-terminal tail of STING was deleted, diABZi still induced LRRK2 activation, suggesting that TBK1/IKKε signaling was not required (Fig. 6 *C*). These results indicate that noncanonical autophagy, in particular the lipidation of GABARAPs, is required for STING-induced LRRK2 activation.

STING-induced noncanonical autophagy is evolutionarily conserved, dating back to early animals such as sea anemone. We therefore tested whether STING activation of TFEB and LRRK2 is also evolutionarily conserved. Interestingly, human STING (*hSTING*), mouse STING (*mSTING*), and FLAG-tagged sea anemone STING (*NvSTING*) all activated both TFEB and LRRK2 when stimulated with a corresponding STING agonist (Fig. 6 *C*). This prompted us to investigate potential interplays between the activation of TFEB and LRRK2 by STING. We found that the activation of TFEB by STING was unaffected by the expression levels of LRRK2 or treatment with the LRRK2 inhibitor Mli-2, suggesting that LRRK2 is dispensable for TFEB activation by STING (Fig. 6*A*). Similarly, FNIP1/2 deficiency did not affect STING-induced Rab10 phosphorylation (Fig. 6*B*), indicating that the FNIP/FLCN/mTORC1 complex is not involved in LRRK2 activation. Taken together, STING-mediated activation of TFEB and LRRK2 appears to be uncoupled although both require GABARAPs lipidation.

### Noncanonical Autophagy Is Required for ALIX-mediated ESCRT Machinery Recruitment to Mitigate Endolysosomal Perturbation.

**Fig. 7.**
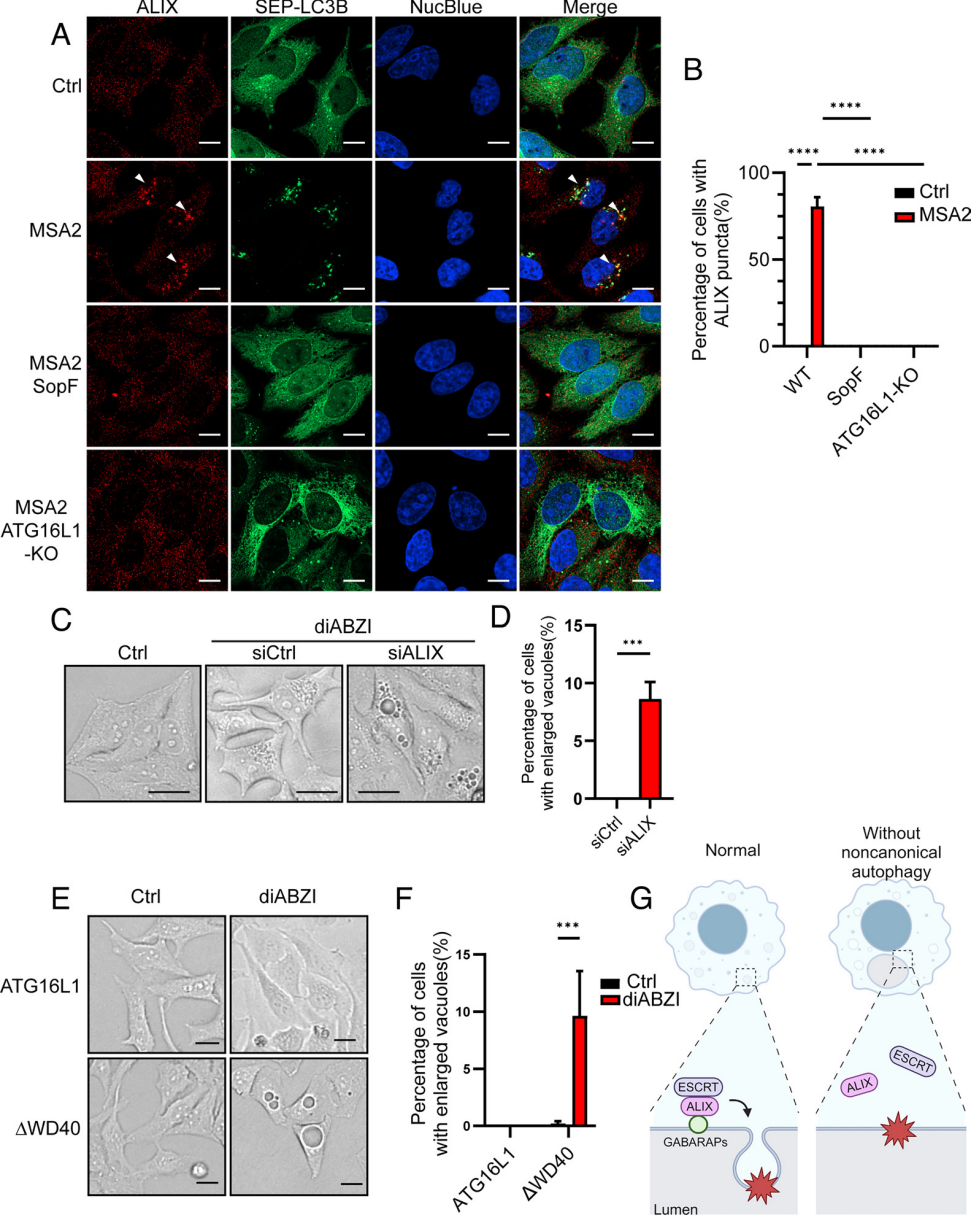
STING-induced autophagy recruits the ALIX–ESCRT to mitigate endolysosomal perturbation. (*A*) HeLa-STING, HeLa-STING-SopF, and HeLa-STING-ATG16L1KO cells that stably express SEP-LC3B were treated with 30 µM MSA2 for 2 h or untreated. Cells were imaged by microscopy using an antibody against ALIX and other markers as indicated. (Scale bar, 10 µm.) Arrows indicate ALIX puncta. (*B*) Quantification of 32 to 70 cells per group represented by *A*. Error bars represent SD. Tested by Tukey’s multiple comparisons test. ****P < 0.0001. (*C*) HeLa-STING cells transfected with siRNA against ALIX or control siRNA were treated with 50 nM diABZI for 24 h, and the morphology of the cells was analyzed by bright-field microscopy. (Scale bar, 25 µm.) (*D*) Quantification of 228 and 214 cells per group shown in *C*. Error bars represent SD. Tested by Tukey’s multiple comparisons test. ***P < 0.001. (*E*) HeLa-STING-ATG16L1 and HeLa-STING-ATG16ΔWD40 cells treated with 50 nM diABZI for 24 h or untreated, then cells were analyzed by bright-field microscopy. (Scale bar, 25 µm.) (*F*) Quantification of more than 300 cells per group shown in E. Error bars represent SD. Tested by unpaired Student’s *t* test. ***P < 0.001. (*G*) A Diagram showing that GABARAPs lipidation facilitates ALIX-mediated ESCRT recruitment which mitigates endolysosomal perturbation.

Since STING trafficking induces endolysosomal membrane perturbation and noncanonical autophagy, we investigated whether noncanonical autophagy plays a role in lysosomal membrane repair. In response to membrane damage, the protein ALIX is recruited to the perturbed membrane to mediate the assembly of the ESCRT complex, which performs the repair of the damaged membrane (26–28). We found that MSA2 treatment led to the recruitment of ALIX and the ESCRT-III protein CHMP1A to the SEP-LC3B puncta (Fig. 7 *A* and *B* and SI Appendix, Fig. S3 A and B); such recruitment was abolished by ectopic expression of SopF or by ATG16L1 KO, suggesting that noncanonical autophagy is required for ALIX-mediated recruitment of the ESCRT machinery (Fig. 7 *A* and *B* and SI Appendix, Fig. S3 A and B). Moreover, ALIX recruitment by treatment with the lysosome-disrupting agent Leu-Leu-O-Methyl (LLOMe, SI Appendix, Fig. S4 A and B) or diABZI (SI Appendix, Fig. S4 C and D) is dependent on GABARAPs. Activation of STING by diABZI in HeLa cells in which ALIX was knocked down by siRNA led to the formation of enlarged vacuoles, a hallmark of endolysosomal perturbation (Fig. 7 *C* and *D*). Importantly, HeLa cells expressing ATG16DWD40 exhibited more enlarged vacuoles in response to diABZI treatment than HeLa-ATG16L1 cells (Fig. 7 *E* and *F*), suggesting that noncanonical autophagy plays an important role in mitigating endolysosomal perturbation (Fig. 7*G*).

## Discussion

Although much is known about how cGAS activation leads to activation of STING and subsequent activation of TBK1 and IRF3 to induce type-I interferons, how STING activates autophagy, and the physiological roles of STING-induced autophagy remain poorly understood. Recent studies, including data presented in this study, have shown that STING trafficking from the ER to Golgi is essential for the conjugation of the LC3/GABARAP family of ubiquitin-like proteins on STING vesicles in a process that does not require key proteins that drive the canonical autophagy process including the ULK1 protein kinase complex and VPS34 lipid kinase complex (9, 11). The mechanism by which STING trafficking induces noncanonical autophagy is still not well understood. Two recent studies suggest that STING functions as a proton channel that causes proton efflux from the Golgi or endosomes (37, 38). Our data suggest that despite significant pH elevation in the Golgi, lipidation of LC3 only occurred on the Golgi-derived vesicles. It is possible that this disturbance of Golgi-derived vesicles or endosomes is “sensed” by the V-ATPase, which then recruits ATG16L1 and other ATG proteins to catalyze the conjugation of LC3/GABARAP family of proteins to the lipid membrane (SI Appendix, Fig. S5). In support of this model, the STING agonist C53, which binds to the putative proton channel, inhibits STING-induced LC3 lipidation. However, C53 also inhibits STING trafficking, so it is not clear whether the inhibition of LC3 lipidation by C53 is due to the blockade of the proton channel or inhibition of STING trafficking. Interestingly, we found that C53 induces rapid aggregation of STING on the ER membrane and that C53 activation of TBK1 and IRF3 is refractory to inhibition by BFA or GCA, indicating that membrane trafficking is not required for STING activation of TBK1 or IRF3 in this case. Thus, aggregation of STING can bypass the requirement of STING trafficking from the ER to Golgi or Golgi-derived endosomes to activate TBK1 and IRF3. However, STING aggregation on the ER cannot bypass the requirement of STING trafficking to induce noncanonical autophagy.

We have previously shown that STING-induced autophagy precedes STING-induced activation of TBK1 and production of type-I interferons, suggesting that autophagy induction is a primordial function of STING during evolution (9). Although STING-induced autophagy plays a role in the clearance of DNA and intracellular pathogens from the cytosol, other physiological functions of STING-induced autophagy have not been explored. Here, we show that STING-induced autophagy leads to activation of the MiT/TFE family of transcription factors, which are known to regulate lysosome biogenesis. We found that STING-induced lipidation of GABARAPs, but not LC3s, recruits FNIP1/2, which form a complex with FLCN, a GAP protein required for mTORC1 activation. Thus, lipidated GABARAPs sequester the FNIP/FLCN complex from mTORC1, thereby preventing mTORC1 from phosphorylating MiT/TFE. The dephosphorylated MiT/TFE transcription factors then translocate to the nucleus to turn on genes important for lysosome biogenesis and autophagy (SI Appendix, Fig. S5).

Another important function of STING-induced autophagy is to activate LRRK2, which also regulates lysosome homeostasis (SI Appendix, Fig. S5). We found that STING-induced nonconical autophagy is required for LRRK2 to phosphorylate the Rab10 GTPase, which maintains endolysosomal homeostasis (23–25). The exact mechanism by which STING-GABARAPs mediates LRRK2 activation needs further investigation. Hyperactivation or chronic activation of LRRK2 has been associated with Parkinson’s disease and Crohn’s disease (50–53). Our results suggest that STING may play a role in these diseases through regulation of LRRK2 activity, which depends on GABARAPs lipidation.

The third function of STING-induced autophagy uncovered through this study is the recruitment of ALIX and ESCRT complex to the STING vesicles (SI Appendix, Fig. S5). The ALIX–ESCRT machinery has been shown to mediate membrane damage repair (26–28). We found that STING stimulation led to the recruitment of ALIX and the ESCRT-III protein CHMP1A to the LC3-conjugated vesicles in a manner that depended on the noncanonical function of ATG16L1. In cells lacking ALIX or the WD40 domain of ATG16L1, STING agonists induce the formation of very large vacuoles that are indicative of membrane damage. These results suggest that STING-induced autophagy plays an important role in recruiting the ESCRT complex to repair membrane damage.

In summary, our results show that STING-induced autophagy activates three pathways that regulate lysosome homeostasis. The activation of the MiT/TFE family of transcription factors regulates lysosome biogenesis, whereas activation of LRRK2 and the recruitment of the ALIX–ESCRT complex mitigate the endolysosomal membrane disruption. Failure to coordinate activation of these three pathways downstream of STING may compromise lysosome homeostasis and lead to human diseases. Unlike TBK1, IRF3, and the type-I interferon pathway which evolved later in vertebrate animals, the MiT/TFE transcription factors, LRRK2, and ESCRT complexes are conserved in metazoans (54, 55). Our results suggest that regulation of lysosome homeostasis by noncanonical autophagy is an evolutionarily conserved function of the cGAS–STING pathway.

## Materials and Methods

### Chemicals, Guide RNA, siRNA, and Antibodies.

The catalog numbers and vendors for chemicals are shown as the following: MSA2 (Tocris, 7353), diABZI (Cayman, 34082), C53 (Cayman,37354), Torin1 (Tocris, 296970), LLOMe (Bachem, 4000725.0001), bafilomycin A1 (Invivogen, tlrl-baf1), BFA (Invivogen, inh-bfa), GCA (EMD Millipore, 345862), Lysotracker Red (Invitrogen L7528), and pHrodo Red Dextran(P10361).

Sequences of guide RNAs for generation of KO cell lines: FIP200 (5′-GGTGTTGAATAGCAATCTT-3′), LC3A (5′- CAGACCGGCCTTTCAAGCAG -3′), LC3B (5′- GTGATAATAGAACGATACAA -3′), LC3C (5′- AAAGTTCCCCAACAAAATCC -3′), GABARAP (5′- GGATCTTCTCGCCCTCAGAG -3′), GABARAPL1 (5′- AGAGAAGGCTCCAAAAGCCA -3′), GABARAPL2 (5′- GGTTCCATCTGATATCACTG -3′), TBK1 (5′- GAAGAACCTTCTAATGCCTA -3′), IKKε (5′- TTTCAGGGCGTGTTGGGCGC -3′), FNIP1 (5′- TCTGGCTTACAATGATGTCG -3′), FNIP2 (5′- TTCCGATGTCAACATGTTAG -3′), and ATG16L1 (5′- GCAGCAAGTGACATGTCGTC -3′).

Sequences of siRNAs used: ALIX (5′-CCUGGAUAAUGAUGAAGGA[dT][dT]-3′).

Sequences of primers used in real-time PCR: 18S (5′- GCAGAATCCACGCCAGTACAAG -3′, 5′- GCTTGTTGTCCAGACCATTGGC -3′), CTSB (5′- GAGCTGGTCAACTATGTCAACA -3′, 5′- GCTCATGTCCACGTTGTAGAAGT -3′), CTSD (5′- GCAAACTGCTGGACATCGTTG -3′, 5′- GCCATAGTGGATGTCAAACGAGG -3′), and GPNMB (5′- GATGCCAAAAGGAAGATGCC -3′, 5′- CTCTGACCATGCTGTCCAGTT -3′).

Antibodies for LC3A/B (#12741), TBK1 (#3504), p-TBK1 (#5483), ATG16L1 (#8089), GAPDH (#5174), TFEB (#4240 for western blot, #37785 for immunofluorescence), STING (#13647), GABARAP (#13733), GABARAPL1 (#26632), GABARAPL2 (#14256), P-S6 (#4858), p-4EBP1 (#2855), IKKε (#3416) and p-IRF3 (#37829)) Rab10 (#8127) were purchased from Cell Signaling Technology. The antibody for STING immunofluorescence (AF6516) was purchased from R&D systems. Antibodies for ATP6V1A (ab199326), ATP6V1D (ab157458), FNIP1 (ab134969), LRRK2 (ab133518), and pRab10 (ab241060) were purchased from Abcam. Antibody for FNIP2(NBP3-12667) was purchased from Novusbio, ALIX (#634501) from Biolegend, and CHMP1A (sc271617) from Santa Cruz Biotechnology.

### Cell Culture.

HeLa, HEK293T, and BV-2 cells were maintained in Dulbecco’s modified Eagle’s medium (DMEM) (Gibco) supplemented with 10% fetal bovine serum (Sigma) and 1% penicillin and streptomycin. THP-1 cells were cultured in RPMI-1640 medium (Gibco) supplemented with 10% fetal bovine serum, 0.05 mM 2-mercaptoethanol (Gibco), and 1% antibiotic-antimycotic (Gibco). All cells were cultured in 37 °C humidified incubators with 5% CO2. Cells were tested as *mycoplasma* negative.

### CRISPR KO Cell Line Generation.

sgRNAs were cloned into lentiCRISPR v2 vectors [Addgene #98290, #98291, #98292, #98293, gifts from Brett Stringer (56)]. Lentivirus was generated with pMD2.G, and psPAX.2 in 293 T cells. The supernatant containing the virus was filtered with 0.45uM filters and then transduced into cells. Selection was performed with puromycin, blasticidin, neomycin, or hygromycin based on the vectors. Single clones were isolated, and the efficiency of KO was validated by western blotting, functionality test, and Sanger sequencing.

### Immunofluorescence, Confocal Microscopy, and Live Cell Imaging.

For immunofluorescence imaging, cells seeded in 8-chambered slides with a 1.5 mm glass bottom (Lab-TekII) were first fixed with 4% paraformaldehyde (PFA) in PBS for 10 min. After three PBS washes, cells were permeabilized with 0.3% Triton-X100 in PBS for 15 min. Alternatively, cells were fixed and permeabilized with ice-cold methanol for 15 min. After PBS washes, cells were blocked with 5% goat serum in PBS for 30 min, followed by staining with primary antibodies in blocking solution for 1 h at room temperature (RT). After rinsing with PBS, cells were stained with goat secondary antibodies and NucBlue (Invitrogen) in blocking solution for 1 h at RT. After three PBS washes, chambered slides were loaded with fresh PBS before microscopic analysis. For live cell imaging, cells were seeded in 8-chambered slides with 1.5 mm glass bottom (Lab-TekII) at approximately 2 × 10^4^ cells per chamber and cultured for 24 h. The live-cell imaging incubator was equilibrated to 37 °C, 5% CO_2_ before loading of chambered slides. All images were acquired using a ZEISS LSM980 confocal microscope with Airyscan2 superresolution module.

### Immunoprecipitation for Detection Of ATG16L1 and V-ATPase Interaction.

HeLa-STING-ATG16L1-KO cells stably expressing FLAG-ATG16L1 or FLAG-ΔWD40 were treated with or without 30 µM MSA2 for 1.5 h. Cells were harvested with a scrapper in cold PBS. After centrifugation at 200×*g* for 3 min, cells were resuspended in three times packed cell volumes of lysis buffer [25 mM Tris-HCl pH7.5, 150 mM NaCl, 2 mM EDTA, 0.8% C12E9, complete protease inhibitor cocktail (Roche), and PhosSTOP (Roche)]. Cells were lysed by pushing through a 27G syringe needle 10 times. After centrifugation at 20,000×*g* for 15 min, the supernatant was incubated with anti-FLAG M2 affinity beads (Millipore) at 4 °C for 1 h. The M2 beads were washed five times with wash buffer (25 mM Tris-HCl pH7.5, 150 mM NaCl, 2 mM EDTA, 1% Triton X-100, and 0.1% C12E9) and then resuspended in 1.2 × SDS sample buffer for western blotting analyses.

### qRT-PCR.

RNA was isolated from cells using the Quick-RNA Miniprep Kit (Zymo Research). iScript™ cDNA Synthesis Kit and iTaq Universal SYBR Green Supermix (Bio-Rad) were used for qRT-PCR analysis according to the manufacturer’s instructions.

### Statistical Analysis and Graphical Illustrations.

Statistical analysis for each experiment was performed in PRISM (GraphPad). Replicates, significance values, and tests performed are indicated in the figure legends. Graphical illustrations were created using BioRender.

## Supplementary Material

Appendix 01 (PDF)

Movie S1. HeLa-STING cells stably expressing SEP (green)-mKate2-TGN46 and miRFP670-LC3 (red) were stimulated with 1 μM diABZI followed by live cell imaging.

Movie S2. HeLa cells stably expressing STING-mGFP (green) and mCherry-Sec61B (red) were stimulated with 1.25 μM C53 followed by live cell imaging.

## Data Availability

All study data are included in the article and/or supporting information.
