# Supplementary material for: STING-induced noncanonical autophagy regulates endolysosomal homeostasis
Source: Proc Natl Acad Sci U S A. 2025 Feb 21;122(8):e2415422122. doi: 10.1073/pnas.2415422122 (PMC11874320; doi:10.1073/pnas.2415422122)
Supplement: Supplementary file 1 — Appendix 01 (PDF) [file pnas.2415422122.sapp.pdf]

PNAS

Supporting Information for

STING-induced noncanonical autophagy regulates endolysosomal homeostasis.

Tuozhi Huang[1, 2#], Chenglong Sun[1, 2#], Fenghe Du[1,2,4] and Zhijian J. Chen[1, 2, 3*]

[#] These authors contribute to the study equally

*Zhijian J. Chen.
Email: zhijian.chen@utsouthwestern.edu.

This PDF file includes:

Figures S1 to S5

Legends for Video S1 and S2

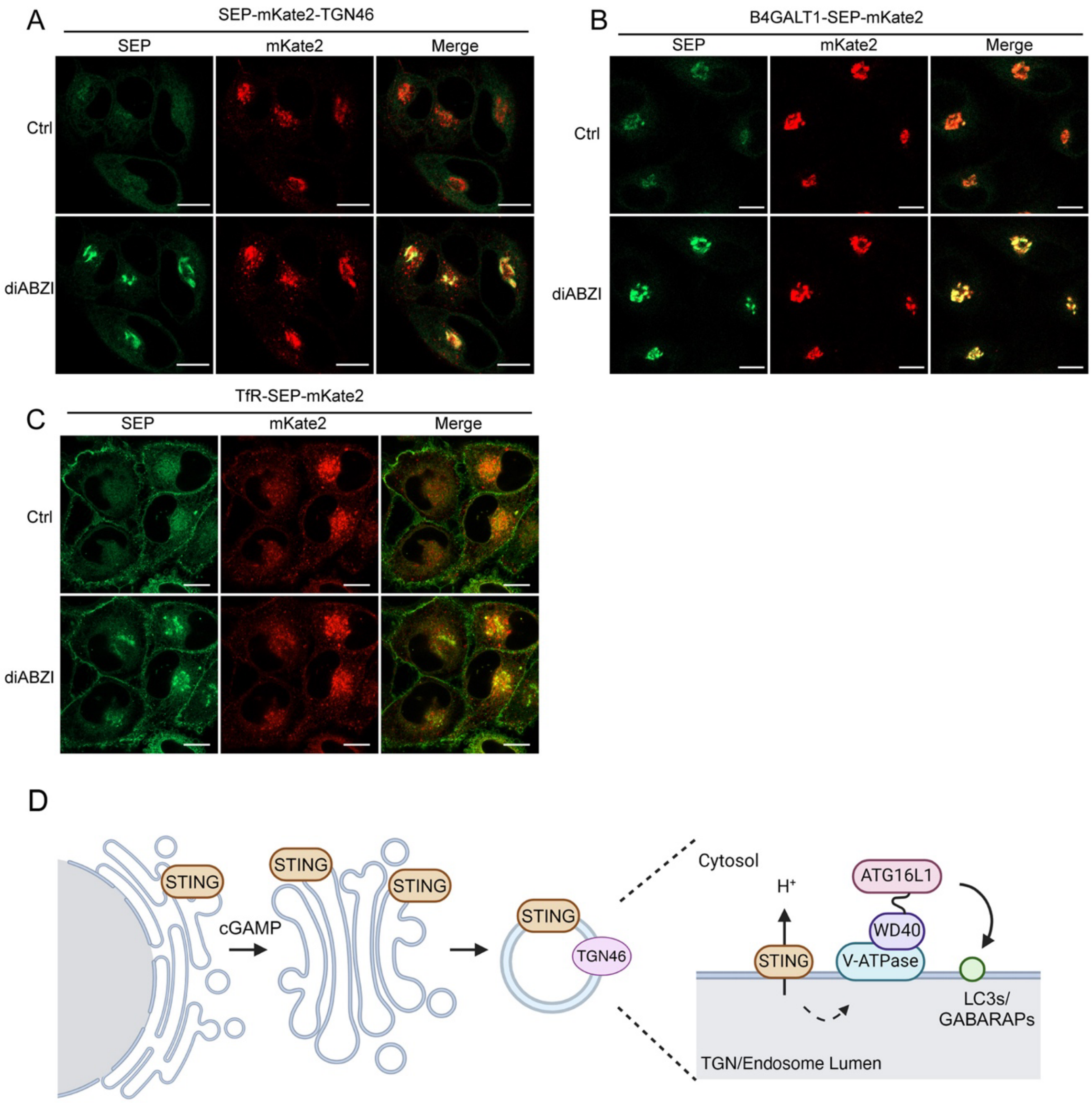

Figure S1. STING activation raises the pH in the Golgi and Golgi-derived vesicles. (A) HeLa-STING cells stably expressing SEP-mKate2-TGN46 were treated with 1 µM diABZI, and cells were analyzed by live cell microscopy. Representative images taken at 0 min (Ctrl) and 30 min (diABZI) are shown. scale bars: 10 µm. (B) &(C) Similar to (A) except that B4GALT1-SEP-mKate2 or TfR-SEP-mKate2 was stably expressed in HeLa-STING cells, respectively. (D) A model depicting how STING trafficking from the ER to Golgi leads to an increase of pH in the Golgi and Golgi-derived endosomes on which V-ATPase recruits ATG16L1, leading to the conjugation of LC3s and GABARAPs onto the lipid membranes.

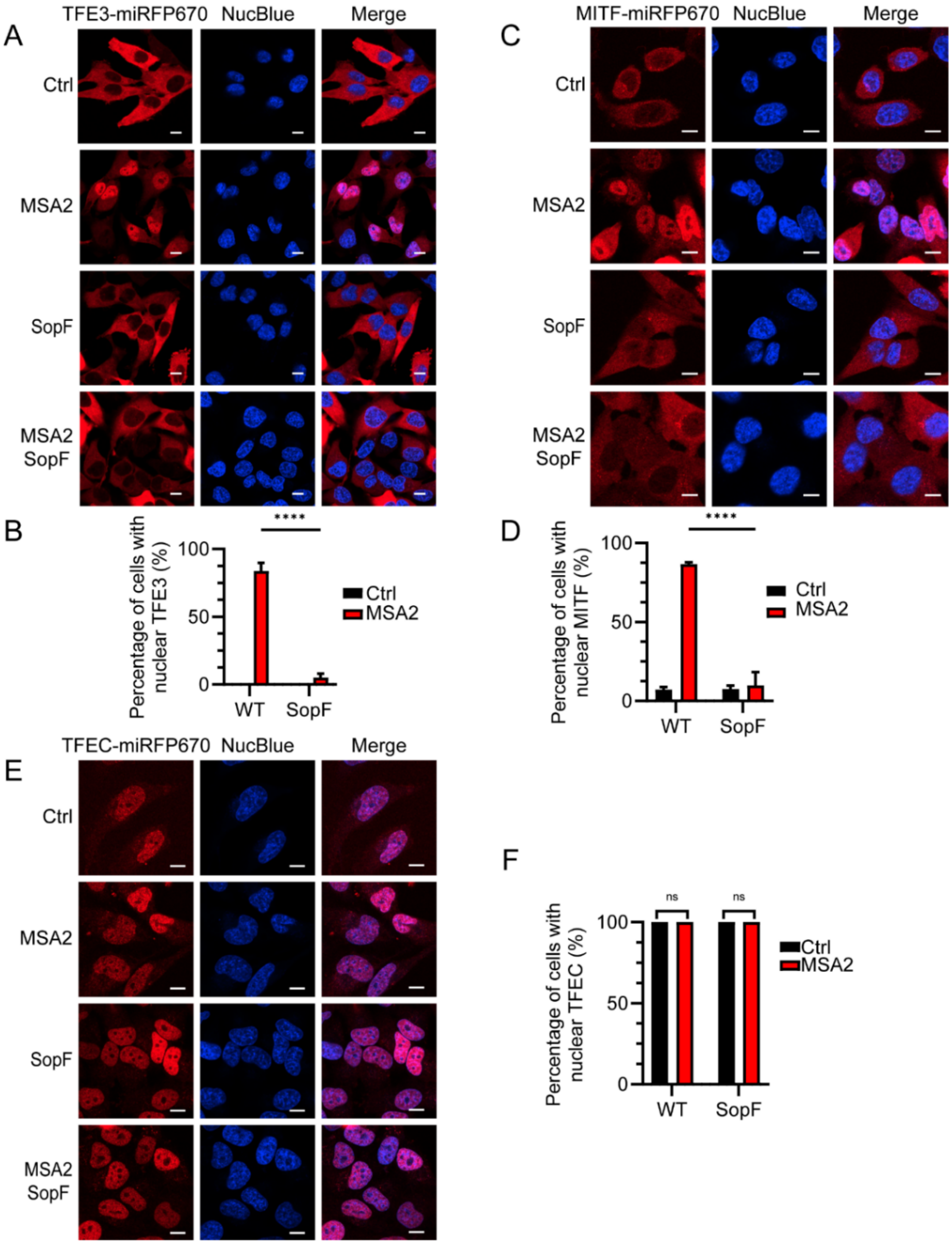

Figure S2. Subcellular localization of TFE3, MITF, and TFEC during STING activation
(A) HeLa-STING and HeLa-STING-SopF cells stably expressing TFE3-miRFP670 were treated with 30 µM MSA2 for 2 h or left untreated, and live cells were imaged by confocal microscopy. scale bars: 10 µm. (B) Quantification of 60-142 cells per group as represented by A. Error bars represent SD. Tested by Tukey's multiple comparisons test. ****P < 0.0001. (C) Similar to (A) except that the cell lines stably expressed MITF-miRFP670. (D) Quantification of 57-105 cells per group as represented by C. Error bars represent SD. Tested by Tukey's multiple comparisons test. ****P < 0.0001. (E) Similar to (A) except the cell lines stably expressed TFEC-miRFP670. (F) Quantification of 57-160 cells per group as represented by E. Error bars represent SD. Tested by Tukey's multiple comparisons test.

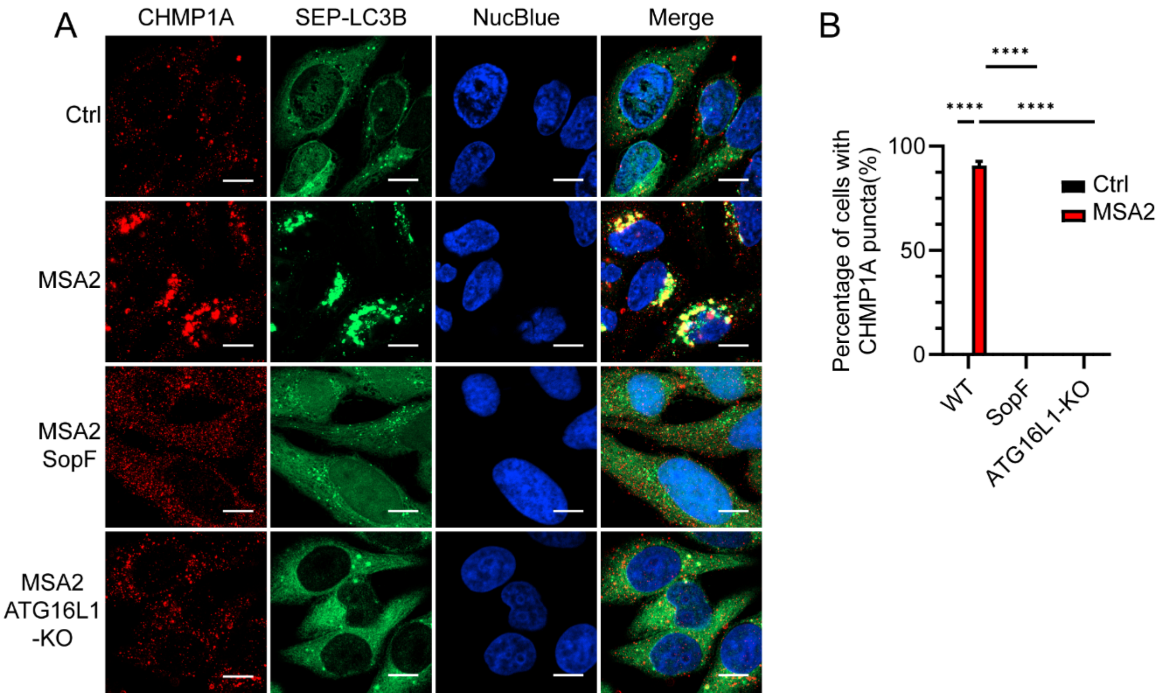

Figure S3. STING-induced noncanonical autophagy is required for CHMP1A recruitment to vesicles. (**A**) HeLa-STING, HeLa-STING-SopF, and HeLa-STING-ATG16L1KO cells that stably express SEP-LC3B were treated with 30 µM MSA2 for 2 h or left untreated. Cells were imaged by microscopy using an antibody against CHMP1A and other markers as indicated. Scale bars: 10 µm. (B) Quantification of 27-50 cells per group as represented by A. Error bars represent SD. Tested by Tukey's multiple comparisons test. ****$P < 0.0001$.

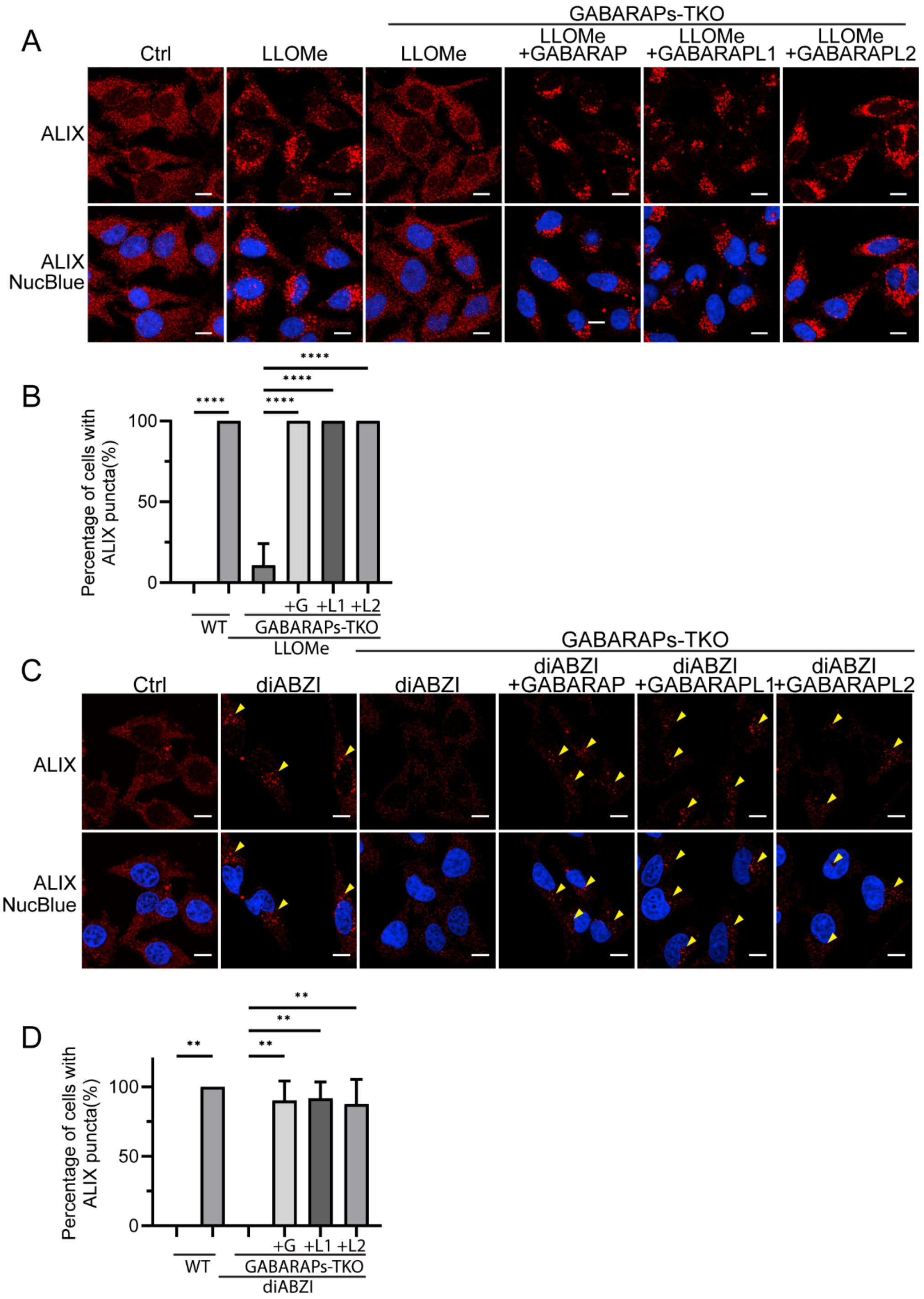

Figure S4. Endolysosomal membrane perturbation by LLOMe or STING agonist induces ALIX foci in a manner that depends on GABARAPs. (**A**) HeLa-STING, HeLa-STING-GABARAPs-TKO cells, HeLa-STING-GABARAPs-TKO cells rescued with GABARAP, GABARAPL1, or GABARAPL2 as indicated were treated with 1 mM LLOMe for 1 h or left untreated. Cells were analyzed by confocal microscopy using antibody against ALIX. The merged images show both ALIX and nuclear DNA staining by NucBlue. scale bars: 10 μm. (**B**) Quantification of 19-45 cells per group as represented by **A**. Error bars represent SD. Tested by Tukey's multiple comparisons test. ****P < 0.0001. (**C**) Similar to (**A**) except that cells were treated with 100 nM diABZI for 2 h, scale bars: 10 μm. Arrows indicate ALIX puncta. (**D**) Quantification of 10-13 cells per group as shown in **C**. Error bars represent SD. Tested by Tukey's multiple comparisons test. **P < 0.01.

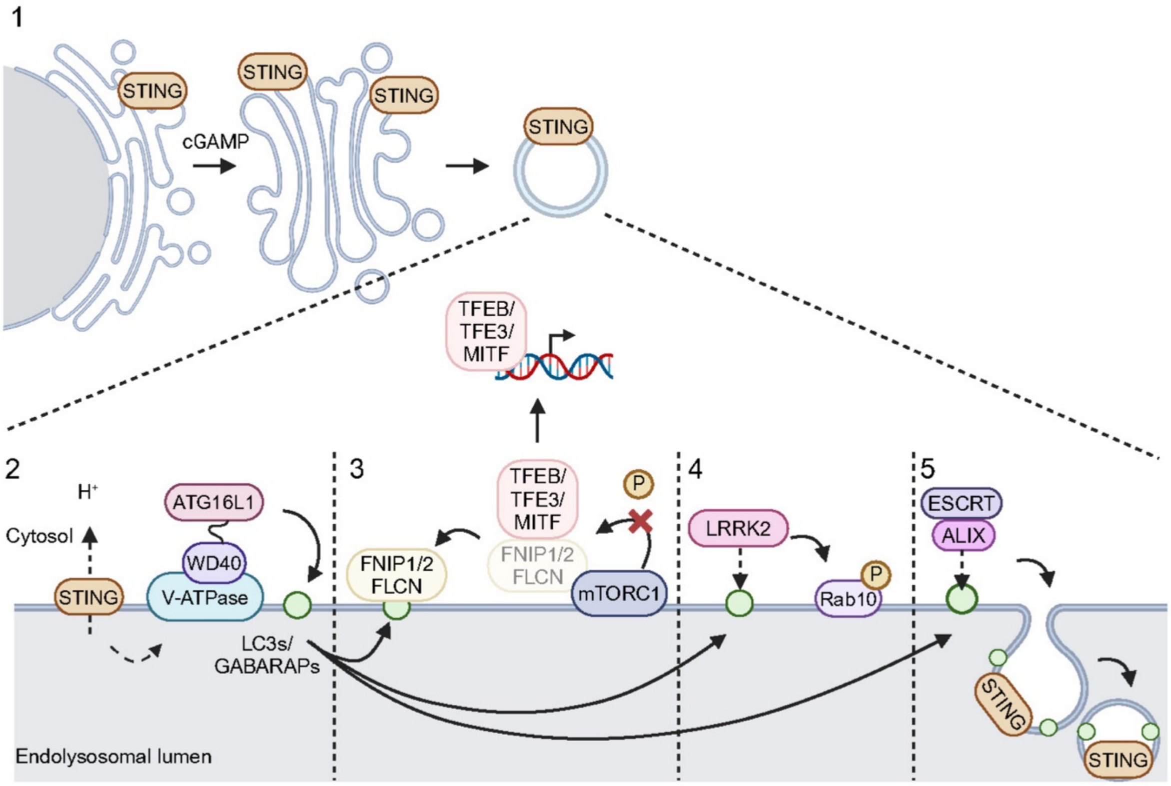

Figure S5. A diagram illustrating the mechanism by which STING induces noncanonical autophagy, leading to endolysosomal biogenesis and repair. 1. Upon binding cGAMP, STING traffics from the ER to the Golgi. 2. STING triggers the elevation of the pH in the Golgi, which likely results in LC3/GABARAPs lipidation on the membrane of Golgi-derived vesicles. 3. GABARAPs binding to FNIP1/2 suppresses mTORC1-mediated phosphorylation of TFEB/TFE3/MITF. The dephosphorylated transcription factors enter the nucleus to turn on the endolysosomal biogenesis program. 4. GABARAPs lipidation activates LRRK2 to phosphorylate Rab10 in response to STING stimulation. 5. GABARAPs lipidation facilitates ALIX-mediated ESCRT recruitment which helps to mitigate endolysosomal perturbation.

Legends for Video S1 and S2:

Supplementary Video S1: HeLa-STING cells stably expressing SEP (green)-mKate2-TGN46 and miRFP670-LC3 (red) were stimulated with 1 μM diABZI followed by live cell imaging.

Supplementary Video S2: HeLa cells stably expressing STING-mGFP (green) and mCherry-Sec61B (red) were stimulated with 1.25 μM C53 followed by live cell imaging.
